# Assessing welfare risks in unowned unsocialised domestic cats in Denmark based on associations with low body condition score

**DOI:** 10.1186/s13028-023-00665-2

**Published:** 2023-01-23

**Authors:** Søren Saxmose Nielsen, Ida Sofie Thuesen, Helena Mejer, Jørgen Steen Agerholm, Stine Thorsø Nielsen, Pikka Jokelainen, Stig Milan Thamsborg, Peter Sandøe

**Affiliations:** 1 Department of Veterinary and Animal Sciences, University of Copenhagen, Grønnegårdsvej 8, 1870 Frederiksberg C, Denmark; 2 Department of Veterinary Clinical Sciences, University of Copenhagen, Højbakkegårds Allé 5A, 2630 Taastrup, Denmark; 3 Infectious Disease Preparedness, Statens Serum Institut, Artillerivej 5, 2300 Copenhagen S, Denmark; 4 Department of Food and Resource Economics, University of Copenhagen, Rolighedsvej 23, 1958 Frederiksberg C, Denmark

**Keywords:** Animal welfare, Body condition, Free-ranging, Health, Parasite, Virus

## Abstract

**Background:**

Populations of unowned unsocialised cats are present worldwide. Generally, there is concern about their welfare. Low body condition score (BCS) is a potentially relevant indicator that is relatively easy to assess: emaciated cats are likely to have welfare problems while thin cats may be at risk of becoming emaciated. The objective of this study was to assess the association of low BSC with a selection of factors relating to the host, disease, and infection in unowned unsocialised domestic cats. We necropsied 598 euthanised unowned unsocialised cats from Denmark. We recorded each cat’s age-group, sex, and neuter status, together with its pregnancy status, the season and location of trapping, as well as gross lesions at necropsy. We also tested for feline immunodeficiency virus and feline leukaemia virus, recorded presence of ectoparasites, and a subsample of the cats were also tested for endoparasites. Cats with no or sparse adipose deposits were categorised as having low BCS, and logistic regression was used to determine the factors associated with low BCS.

**Results:**

Of the cats, 11.4% had low BCS. Season, age-group and sex were associated with low BCS and confounded potential associations with other variables. Intact adult males and females in spring and early summer were at highest risk of low BCS. When these factors were taken into account, cats with biting lice had 2.8 (95% confidence interval (CI) 1.4–5.4) times higher odds of low BCS, and cats with gastro-intestinal findings (i.e., enlarged mesenteric lymph nodes, abdominal hernia, diarrhoea, obstructive foreign bodies, or diaphragmatic hernia) had 50 (95% CI 10–417) times higher odds of low BCS, than cats with no such findings. Cats with low BCS were primarily adult intact cats with tooth lesions and skin lesions, ear mite infection, and positive test result for feline immunodeficiency virus.

**Conclusions:**

The results highlight associations that can be used to define a risk profile: low BCS, notably in summer-autumn, in an unowned unsocialised cat was associated with underlying, less visible problems. Thus, low BCS can be more than a step towards being emaciated; it can also be an indicator of other underlying welfare problems.

## Background

Populations of unowned unsocialised cats are present worldwide. In Denmark there is a population of around 89,000 (95% confidence interval: 67,000–111,000) unowned domestic cats. Of these about a third are considered to be socialised and two-thirds to be unsocialised [1]. A socialised cat is one that in the sensitive period as a kitten has been exposed to humans and therefore typically will be trustful and forthcoming to humans and easy to handle. Unowned socialised cats (stray cats) have been abandoned by, or have wandered away from, their previous owners. These cats may end up in shelters, from where the majority are then housed with new owners [2]. In contrast, unowned cats that are ‘unsocialised’ (sometimes referred to as feral cats [3]) have never lived with humans. They will typically keep a distance to humans and cannot easily be handled even though there is some indication that they with some effort can attach to individual humans [4]. These cats may be offspring of intact unowned socialised cats, some of which (e.g., so-called “barn cats”) live in a grey zone between owned and unowned animals. In a temperate country like Denmark unowned, unsocialised cats usually have some contact with the people living in their vicinity. The cats may even be dependent on people due to feeding or scavenging on anthropogenic food sources [1]. In some countries, large numbers of unowned unsocialised cats are handled through trap-neuter-return (TNR) programmes [5], but in others, like Denmark, TNR plays a limited role. Unowned unsocialised cats may be considered a nuisance for a number of reasons, for example that they are noisy in the night, that they leave their faeces in gardens, that they fight with and scare owned cats, and that they kill birds and other wild animals; consequently as a group they are often unpopular with people [6]. Some unowned unsocialised cats are consequently euthanised. Even people who have concerns about the welfare of this group of cats may be in favour of euthanasia [7] as the best way of preventing unnecessary suffering in cases where it is not an option to place them in a colony of neutered cats under human supervision.

What constitutes good animal welfare is contested. However, there is reasonably wide agreement on the view that ultimately animal welfare is about the feelings of the animals, which is reflected in the influential Five Domains Model of animal welfare [8]. This model, which is also widely applied to wild animals [9], views the feelings of the animals, the so-called mental domain as a function of four other domains: behaviour, environment, health, and nutrition. While behavioural interactions are not normally considered to be an issue in unowned unsocialised cats the three others may be. Some of these domains may be associated. For example, if a cat suffers from injury or disease, it may be unable to feed normally, thus adversely affecting the domain of nutrition, leading to hunger.

Poor body condition, as indicated by a low body condition score (BCS), may be a key indicator of an actual or potential welfare problem [10]. Body condition can be an indicator of how well an unowned unsocialised cat has been coping with disease and its environment, and a trained observer may be able to assess the body condition from the distance, although this has not been methodically assessed.

Few studies have assessed risk factors for poor welfare in stray or unowned unsocialised cats. One study of 188 necropsied stray cats in Western Australia found season and gastro-intestinal helminth biomass to be associated with body condition in males [10]. Apart from this, the available literature is sparse and addresses populations that appear to have included a mixture of socialised and unsocialised unowned cats and provides only limited information about the associations between host factors, diseases and infections with poor body condition or other measures of welfare.

To investigate whether low BCS could serve as a parameter of the risk of reduced welfare in unowned unsocialised domestic cats, we determined the association between BCS and a selection of host factors, diseases and infections. Thereby our focus was on the association between the three most relevant domains concerning the welfare of unowned, unsocialised cats: environment, health and nutrition. More specifically, we looked at how certain states of the environment (season) and health (viral and parasite infections and lesions) associate with nutrition (low BCS).

## Methods

The materials used in this study have been described in detail elsewhere [11]. Here we only provide relevant details of the included cats and the diagnostic procedures. Overall, a macroscopic pathoanatomical examination was performed, including examination for feline immunodeficiency virus (FIV) and feline leukaemia virus (FeLV). Furthermore, we examined for ectoparasites. Lastly, a subset of the cats was examined for a range of endoparasites.

### Included cats

The cats included in the study were all unowned unsocialised domestic cats that had been euthanised. Details on how and by whom the cats were euthanized have been reported previously [11]. The reason why the individual cats were caught and euthanised was not recorded. In Denmark, people can report the presence of unowned cats to their municipalities. After this, the municipalities can, and normally will, give permission for the cat to be trapped and if the cat is unowned, subsequently euthanised. In 2019 when the cats included in this study were trapped, in approximately half of the 98 Danish municipalities, trapping and euthanising was managed by the animal welfare non-governmental organisations (NGOs) Kattens Værn and Dansk Dyreværn Aarhus. Thus, these organisations provided the study materials. For each cat trapped, the NGOs had assessed whether it was owned (ear- or chip-marked and thus recorded in the official cat registries) and socialised (approachable), and if it was it was returned to its owner or put into a shelter for adoption. If there was doubt about whether a cat was socialised or unsocialised, it was brought to a shelter and observed for social behaviour for 1–2 weeks. Cats that were lactating were released irrespective of their degree of socialisation unless the kittens were also found. Only cats that were deemed to be unowned and unsocialised were euthanised and thus eligible to be included in this study. No cats were euthanised for the purpose of this study. Permission to carry out the study was received from the Animal Ethics Institutional Review Board at the Department of Veterinary and Animal Sciences, University of Copenhagen.

After euthanasia, the cats were placed in non-transparent plastic bags and stored at − 20 °C. The origin and characteristics of the cats were blinded to the person selecting cats for inclusion in this study. Generally, we strove to include cats evenly from the following five NUTS-2 (nomenclature of territorial units for statistics) regions: The North Denmark Region, The Central Denmark Region, The Region of Southern Denmark, The Region Zealand, and The Capital Region of Denmark [12]. The sample size (n = 598) and the subsample of cats examined for endoparasites were determined by necropsy capacity and constraints imposed by the costs of laboratory analyses. No formal randomization was done for the endoparasite subsample, but the cats were stored in non-transparent plastic bags, which were shuffled during movement to and from − 80 to − 20 °C freezers (for inactivation of any *Echinococcus* spp.). This process resulted in some level of randomization.

The origin of the cats was recorded by postal code, and further categorised according to NUTS-2 regions. The included cats had been trapped from 15 February to 15 September 2019. This seven-month period was divided into winter/spring (15 February to 31 March), spring/summer (1 April to 31 July) and summer/autumn (1 August to 15 September).

The cats were categorised as ‘juvenile’ if one or more deciduous teeth were observed, and otherwise as ‘adult’. They were also arranged into five reproductive status categories, based on sex, neutering and pregnancy: intact male, neutered male, intact non-pregnant female, pregnant female, and neutered female.

### Diagnostic procedures

The post mortem procedures were described in detail previously [11]. Briefly, the cats were necropsied after thawing, and gross lesions were recorded. One person (IST) did the necropsies, including body condition scoring, and assessment of age and reproductive status, along with sampling for further laboratory analyses. Necropsy findings were grouped according to organ system, except for the gastro-intestinal (GI) system, where tooth lesions were recorded separately. Each cat was initially given one of five BCSs: emaciated, thin, normal, moderately overweight or obese [11]. Analyses for FIV and FeLV were conducted using polymerase chain reaction (PCR) on a sample of bone marrow in a commercial laboratory (Laboklin, Bad Kissingen, Germany). No diagnostic sensitivity and specificity data are available for these methods; however, given that they are both PCR-based, and bone marrow was used as the diagnostic specimen, we expected based on expert knowledge > 80% and > 99% for diagnostic sensitivity and diagnostic specificity, respectively.

Ectoparasite infestations were determined following a previously described procedure [11] to identify lice (*Felicola subrostratus*), fleas (*Ctenocephalides felis*, and fleas belonging to the subfamily Ceratophyllinae), ticks (*Ixodes ricinus*) and ear mites (*Otodectes cynotis*). Briefly, endoparasitic diagnoses focused on intestinal helminths (e.g., *Toxocara cati*, *Hydatigera taeniaeformis* and *Mesocestoides lineatus*), which were recovered using the sedimentation and counting technique (SCT) (n = 250) [13, 14], and on adults of *Eucoleus aerophilus* and first stage larvae (L1) of *Aelurostrongylus abstrusus*, which were recovered from the bronchi (n = 217) by dissecting and flushing. Faecal samples were examined for helminth eggs [15] and for *Giardia duodenalis* cysts (n = 48) [16]. Muscle tissue from the tongue (n = 512) was examined for *Toxoplasma gondii* by real-time PCR [17]. Most of the data were categorised in count intervals for the purpose of statistical analysis. However, *T. cati* data were categorised by the numbers of juvenile worms, adult worms, worms in total, and eggs, and *E. aerophilus* data were categorised by the numbers of worms and eggs. Examinations for endoparasites were done by separate investigators and blinded to the other necropsy findings.

### Statistical analysesd

In the statistical analyses the groups ‘emaciated’ and ‘thin’ were pooled into a single category ‘low BCS’. The cats in other BCS groups were pooled into a category ‘normal-high BCS’. Descriptive statistics were created by cross-tabulation of the outcome variable (dichotomised BCS-category) and each of the other variables.

Logistic regression was then carried out using the glm()-function in R version 4.1.1 [18] with ‘low BCS’ (yes/no) as outcome variable. The following analytical strategy was employed. Three variables inherent to the cat (season, age-group and reproductive status), along with all their pair-wise interactions, were included in the model first. This model was then reduced using the likelihood ratio test with a cut-off of P > 0.05, by first removing non-significant interaction terms and subsequently other non-significant variables. The variable with the largest P-value was removed first. However, in the reduction process, we also observed if the parameter estimates changed as indication of possible confounding. If the parameter estimates changed more than 20%, even if the variable was non-significant, the variable was retained in the model.

Following the assessment of the inherent variables, all other variables were included in the model, and a backward elimination process was carried out, following the same principles as above, except that due to the risk of committing Type 1 statistical errors, we did not assess the pair-wise interactions for the remaining variables.

The fit of the model was assessed using the likelihood ratio test for the fitted and the empty model and through an estimate of McFadden’s pseudo-R^2^. The latter was estimated using the pR2()-function in the pscl-package in R [19].

## Results

### Population

Regional and seasonal distribution, along with age-group and reproductive status distributions of the 598 necropsied cats, are shown in Table 1. Most cats (90.8%) were intact. No obese cats were recorded; 11.4% of the cats had low BCS. Cats with low BCS seemed to be more common in spring/summer (20%) than in other seasons. One juvenile cat (1%) was recorded as thin, while no juveniles were emaciated. Of the adults, 14% were thin (n = 65) or emaciated (n = 2). None of the neutered cats were thin or emaciated. The seemingly highest proportions of cats with low BCS were observed among the intact males (15.1%) and the pregnant females (15.0%).

**Table 1:** Population characteristics. Distribution of demographical variables of 598 euthanised unowned unsocialised domestic cats sampled in Denmark in 2019, stratified into cats with low or normal-high body condition score

Variables	Categories	Body condition score	
		Normal-high	Low
Region	The North Denmark Region	84 (86%)	14 (14%)
	Central Denmark Region	114 (90%)	13 (10%)
	The Region of Southern Denmark	136 (84%)	26 (16%)
	Region Zealand	153 (912%)	14 (8%)
	The Capital Region of Denmark	42 (100%)	0 (0%)
	Missing information	1 (50%)	1 (50%)
Season	Spring/summer	160 (80%)	41 (20%)
	Summer/autumn	265 (93%)	21 (7%)
	Winter/spring	105 (95%)	6 (5%)
Age-group	Juvenile	111 (99%)	1 (1%)
	Adult	419 (86%)	67 (14%)
Reproductive status	Female, intact, non-pregnant	171 (92%)	14 (8%)
	Female, pregnant	68 (85%)	12 (15%)
	Female, neutered	33 (100%)	0 (0%)
	Male, intact	236 (85%)	42 (15%)
	Male, neutered	22 (100%)	0 (0%)
Total		530 (89%)	68 (11%)

### Descriptive statistics

The distributions of health variables cross-tabulated with the BCS variable are shown in Table 2. Seven cats were not tested for FIV and FeLV; three were not examined for fleas and lice, two were not examined for ear mites, and only a subsample of cats was tested for endoparasites. Altogether, we had complete data for host, health and ectoparasite variables for 584 cats. These data were used in the majority of the logistic regression analyses.

**Table 2:** Descriptive statistics for disease-related factors and low body condition. Cross-tabulation of body condition and selected health variables of 598 unowned unsocialised domestic cats in 2019 in Denmark, stratified according to body condition score

Variables	Categories	Body condition score	
		Normal-high	Low
Tooth lesions	Not present	449 (90.0%)	50 (10.0%)
	Present	81 (81.8%)	18 (18.2%)
Skeletal system lesions	Not present	521 (88.8%)	66 (11.2%)
	Present	9 (81.8%)	2 (18.2%)
Skin lesions	Not present	511 (89.0%)	63 (11.0%)
	Present	19 (79.2%)	5 (20.8%)
Urogenital system lesions	Not present	493 (89.2%)	60 (10.8%)
	Present	37 (82.2%)	8 (17.8%)
GI system findings	Not present	526 (89.9%)	59 (10.1%)
	Present	4 (30.8%)	9 (69.2%)
Liver lesions	Not present	513 (88.9%)	64 (11.1%)
	Present	17 (80.9%)	4 (19.1%)
FIV (PCR)	Negative	479 (89.4%)	57 (10.6%)
	Positive	44 (80.0%)	11 (20.0%)
	Missing	7 (100.0%)	0 (0.0%)
FeLV (PCR)	Negative	519 (88.9%)	65 (11.1%)
	Positive	4 (57.1%)	3 (42.9%)
	Missing	7 (100.0%)	0 (0.0%)

*FIV* feline immunodeficiency virus, *FeLV* feline leukaemia virus, *GI* gastro-intestinal, except teeth, *PCR* polymerase chain reaction

FIV and FeLV positive cats had a seemingly higher risk of low BCS, with 20% and 43% having low BCS, respectively, as compared with 11% cats with low BCS among the test-negative cats for both infections. Cats with low BCS also seemed more likely than cats with normal BCS to have lice (24% vs. 9% for those without lice) or ear mites (22% vs. 10% for those without ear mites) (Table 3). Among the 250 cats examined with the SCT, cats positive for *A. abstrusus* L1, *E. aerophilus* eggs, and high loads of juvenile *T. cati* worms appeared to be more likely to have low BCS, with infection prevalences of 24%, 17%, and 22%, respectively (Table 4). Because these are univariable comparisons, all associations identified at this stage might be subject to multicollinearity.

**Table 3:** Descriptive statistics for ectoparasite infestations and low body condition. Cross-tabulation of body condition and ectoparasite infestations of unowned unsocialised domestic cats in 2019 in Denmark, stratified according to body condition score

Variables	Categories	Body condition score	
		Normal-high	Low
Fleas	Not present	458 (88.6%)	59 (11.4%)
	Present	69 (90.8%)	7 (9.2%)
	Missing	3 (60.0%)	2 (40.0%)
Lice	Not present	454 (91.3%)	43 (8.7%)
	Present	73 (76.0%)	23 (24.0%)
	Missing	3 (60.0%)	2 (40.0%)
Ticks	Not present	505 (89.1%)	62 (10.9%)
	Present	25 (83.3%)	5 (16.7%)
Ear mites	Not present	489 (89.7%)	56 (10.3%)
	Present	39 (78.0%)	11 (22.0%)
	Missing	2 (66.7%)	1 (33.3%)

**Table 4:** Descriptive statistics for endoparasite infections and low body condition. Cross-tabulation of body condition and endoparasite infections of unowned unsocialised domestic cats in 2019 in Denmark

Variables	Count category^a^	Body condition score	
		Normal-high	Low
*Aelurostrongylus abstrusus* first stage larvae	[0,1)	166 (92.2%)	14 (7.8%)
	[1,55000)	28 (75.7%)	9 (24.3%)
	Missing	336 (88.2%)	45 (11.8%)
*Eucoleus aerophilus* eggs	[0,1)	206 (91.2%)	20 (8.8%)
	[1,461)	20 (83.3%)	4 (16.7%)
	Missing	304 (87.4%)	44 (12.6%)
*Eucoleus aerophilus* worms	[0,1)	177 (89.4%)	21 (10.6%)
	[1,7)	17 (89.5%)	2 (10.5%)
	Missing	336 (88.2%)	45 (11.8%)
*Hydatigera taeniaeformis* worms	[0,1)	135 (90.6%)	14 (9.4%)
	[1,38)	91 (90.1%)	10 (9.9%)
	Missing	304 (87.4%)	44 (12.6%)
*Mesocestoides lineatus* worms	[0,1)	217 (90.0%)	24 (10.0%)
	[1,51)	9 (100.0%)	0 (0.0%)
	Missing	304 (87.4%)	44 (12.6%)
*Toxocara cati* eggs	[0,1)	50 (90.9%)	5 (9.1%)
	[1,1000)	54 (87.1%)	8 (12.9%)
	[1000,3000)	59 (89.4%)	7 (10.6%)
	[3000,27300)	63 (94.0%)	4 (6.0%)
	Missing	304 (87.4%)	44 (12.6%)
*T. cati* adult worms	[0,3]	83 (90.2%)	9 (9.8%)
	(3,11]	70 (88.6%)	9 (11.4%)
	(11,122]	73 (92.4%)	6 (7.6%)
	Missing	304 (87.4%)	44 (12.6%)
*T. cati* juvenile worms	[0,1)	118 (90.8%)	12 (9.2%)
	[1,10)	90 (92.8%)	7 (7.2%)
	[10,100)	18 (78.3%)	5 (21.7%)
	Missing	304 (87.4%)	44 (12.6%)
*T. cati* worms, total	[0,1)	23 (85.2%)	4 (14.8%)
	[1,10)	102 (92.7%)	8 (7.3%)
	[10,20)	39 (88.6%)	5 (11.4%)
	[20,131)	62 (89.9%)	7 (10.1%)
	Missing	304 (87.4%)	44 (12.6%)
*Giardia duodenalis* cysts	[0,1)	32 (84.2%)	6 (15.8%)
	[1,843)	8 (80.0%)	2 (20.0%)
	Missing	490 (89.1%)	60 (10.9%)
*Toxoplasma gondii* DNA	Negative	220 (87.3%)	32 (12.7%)
	Positive	236 (91.1%)	23 (8.9%)
	Missing	74 (85.1%)	13 (14.9%)

^a^Categories based on counts, i.e. the first category includes 0 only, while others give the range of the counts observed, e.g. [1,55000] refers to a range of 1 to 55,000

### Analytical statistics

The results of the univariable logistic regression analyses for the five significant variables (season, age-group, reproductive status, lice and GI findings) are shown in Table 5, and results of the multivariable logistic regression are shown in Table 6. In the latter, all inherent variables (season, age-group, reproductive status) were statistically associated with low BCS and were thus retained in the model. No pair-wise interactions between season, age-group and reproductive status were found. Lice and findings in the GI system were also included as statistically significant. The resulting McFadden’s pseudo-R^2^ was 0.26.

**Table 5:** Univariable results from analytical statistics.Odds ratios (OR) with 95% confidence intervals (CI) from univariable logistic regression analyses including variables that were associated with low body condition score in 598 necropsied unowned unsocialised domestic cats in Denmark

Variable	Category	Odds ratio	Std. error	Lower 95% CI	Upper 95% CI
Season	Winter/spring	1		Reference	
	Spring/summer	4.4	0.5	1.9	11.9
	Summer/autumn	1.4	0.5	0.6	3.9
Age-group	Juvenile	1			
	Adult	17.5	1.0	3.8	311
Reproductive status	Female, intact, non-pregnant	1	Reference		
	Female, pregnant	2.0	0.4	0.9	4.6
	Female, neutered	0.0	1072	0.0	INF
	Male, intact	2.0	0.3	1.1	3.9
	Male, neutered	0.0	1304	0.0	INF
Lice	No	1	Reference		
	Yes	3.4	0.3	1.9	5.9
Gastrointestinal findings^a^	Absent	1	Reference		
	Present	20.3	0.6	6.4	76.8
Feline immuno-deficiency virus	Negative	1	Reference		
	Positive	1.9	0.4	0.9	3.9
Skin lesions^a^	Absent	1	Reference		
	Present	2.3	0.5	0.7	5.9
Tooth lesions^a^	Absent	1	Reference		
	Present	2.1	0.3	1.1	3.7
Ear mites	Absent	1		Reference	
	Present	2.5	0.4	1.2	5.1
*Aelurostrongylus abstrusus* first stage larvae	Absent	1	Reference		
	Present	4.0	0.5	1.5	10.2
*Hydatigera taeniaeformis* worms	Absent	1	Reference		
	Present	1.0	0.4	0.4	2.3
*Toxocara cati* eggs	Absent	1	Reference		
	[1,1000)	1.5	0.6	0.5	5.1
	[1000,3000)	1.0	0.6	0.3	3.7
	[3000,27300)	0.6	0.7	0.2	2.6

^a^Gastro-intestinal findings: diarrhoea, enlarged mesenteric lymph nodes, obstructive foreign bodies, abdominal hernia, diaphragmatic hernia; teeth lesions: missing or damaged teeth; skin lesions: ulcerations, bites, othaematoma

**Table 6:** Multivariable results from analytical statistics. Odds ratios (OR) with 95% confidence intervals (CI) from multivariable logistic regression analyses including variables that were associated with low body condition score in 598 necropsied unowned unsocialised domestic cats in Denmark

Variable	Category	Odds ratio	Std. error	Lower 95% CI	Upper 95% CI
Intercept	Juvenile/Intact female/winter-spring/No other findings	1	Reference		
Season	Spring/summer	4.0	0.5	1.6	11.4
	Summer/autumn	1.1	0.5	0.4	3.2
Age-group	Adult	30.6	1.1	5.6	598
Reproductive status	Female, pregnant	1.2	0.5	0.4	3.0
	Female, neutered	0.0	957.7	0.0	∞
	Male, intact	1.9	0.4	0.9	4.2
	Male, neutered	0.0	1263.9	0.0	∞
Lice	Present	2.8	0.3	1.4	5.4
Gastrointestinal (GI)	Findings^a^	49.5	0.9	9.5	417
Teeth	Lesions^a^	1.1	0.4	0.5	2.3
Skin	Lesions^a^	0.7	0.7	0.2	2.3
Feline immuno-deficiency virus (FIV)	Positive	1.1	0.4	0.4	2.5
Ear mites	Present	2.0	0.4	0.8	4.7

^a^Findings included: gastro-intestinal: diarrhoea, obstructive foreign bodies, enlarged mesenteric lymph nodes, abdominal hernia, diaphragmatic hernia; teeth: missing or damaged teeth; skin: ulcerations, bites, othaematoma

Among the remaining variables, it was found that age-group confounded the parameter estimates of FIV, skin lesions, presence of ear mites, *H. taeniaeformis* worms and *A. abstrusus* L1. Reproductive status confounded findings related to teeth, FIV and *T. cati* eggs. Consequently, the variables FIV, skin lesions, tooth lesions, and ear mites were forced into the model. However, the endoparasite variables were presented only as univariable results, because the dataset including these was much smaller as a result of many missing observations.

None of the remaining variables were associated with low BCS when taking season, age-group and reproductive status into account.

The primary factors associated with low BCS were adult age-group (odds ratio (OR): 17; 95% confidence interval (CI) 3.8–311), GI findings (diarrhoea, enlarged mesenteric lymph nodes, obstructive foreign bodies) (OR: 20; 95% CI 6.4–77), spring/summer season (OR: 4.4; 95% CI 0.5–3.9), lice (OR: 3.4; 95% CI 1.9–5.9), and ear mites (OR: 2.0; 95% CI 0.8–4.7). Skin lesions, tooth lesions and FIV-positivity also contributed to the model, but they were confounded by age-group, reproductive status or both. Consequently, they cannot be fully separated, and the odds ratios presented in Tables 5 and 6 differ. There seemed to be little effect of these three variables when corrected for age-group and reproductive status.

*Aelurostrongylus abstrusus* L1, *H. taeniaeformis* worms and *T. cati* eggs were all associated with low BCS, but due to the collinearity effect of age-group or reproductive status could not be separated from these.

## Discussion

This is the first comprehensive study of a wide range of welfare risk factors concerning an unowned unsocialised domestic cat’s environment or health status that can be linked to the animal’s being in a problematic nutritional state in terms of being thin or emaciated (i.e., the complete absence of fat deposits). Two (0.3%) of the 598 necropsied cats were emaciated, and no specific cause of this was identified. Irrespective of cause, emaciation is considered a severe welfare issue for the individual animal, and it seems to be uncommon in unowned unsocialised domestic cats in Denmark. The BCS ‘thin’ was more common (68 cats; 11.0%), but this body condition is much less challenging for the animal than emaciation: the main potential welfare issue it presents is the risk that it might progress to emaciation.

The model pseudo-R^2^ was 0.26, which suggests a reasonably fit, but with additional information not included in the model. Cats trapped in the season spring/summer had the highest odds of low BCS. Also, only one juvenile cat was categorised as thin, and no neutered cats were thin or emaciated. It was intact mature/grown-up males and pregnant females that were primarily affected, which is not surprising considering the nutritional needs of pregnancy and potential roaming of males. The only internal health-related factor associated with low BCS when age-group was controlled for was GI findings, and the only parasites found to be associated with low BCS were lice. However, skin lesions, tooth lesions and the presence of ear mites, FIV and *H. taeniaeformis* worms, *A. abstrusus* L1, and *T. cati* eggs were all associated with low BCS when age-group and reproductive status were not controlled for. Such a finding could be common for age-related conditions, but it is not possible to verify in this study. We have previously determined the prevalence of a number of conditions that unowned unsocialised cats in Denmark suffer from, and have estimated that 17% have major health issues [11]. These include mostly damaged teeth, which has also been reported as a major finding among TNR cats in Japan [20].

Season, age-group and reproductive status were all significant factors, and age-group and reproductive status appeared to confound the effect of some of the other variables. This complicated the interpretation, particularly because the small sample size did not permit stratification into subgroups. It should be noted that lactating cats were excluded from sampling, and inference could not be made on these. One way to overcome confounding is through stratification; however, this would require a larger sample size.

The finding that the adult age-group was associated with low BCS conflicts with an Israeli study of urban TNR cats [21] describing that kittens were more likely to have lower BCS than adults, and that neutered adult cats were more likely to be obese than intact adults. However, because age-group is known to be associated with a number of infections, diseases and conditions, our result makes good sense. The older cats become, the more likely they are to have been exposed to, and acquire, for example, FIV and some parasites, or to have become involved in situations resulting in injuries. Although exposure to some pathogens may lead to immunity, this means there are many conditions from which age-group cannot be separated, which must be considered in the analyses and their interpretation.

Season was found to be associated with low BCS. Cats that had been trapped during spring/summer had the highest odds of low BCS, whereas those trapped during summer/autumn had lower odds, as has also been observed in Australia, although in stray cats [10]. These findings are not surprising given that the availability of feed increases over summer, when the number of prey animals is higher and people spend more time outside and may be more likely to provide feed for cats. It is surprising, however, how little BCS of cats trapped in winter/spring seemed to differ from those trapped in summer/autumn (Table 6), but the peak in low BCS coincides with the breeding season, which may be physiologically demanding for these cats. Reproductive status also displayed associations with BCS: neutered cats had low odds of having low BCS, while intact males and females, as well as pregnant cats, had higher odds (Table 6). This finding is consistent with one study on TNR cats [21], where neutering has been identified as a protective factor against poor body condition. Another study not assessing the potential confounding effects reported that intact male TNR cats are more likely to be injured than intact female TNR cats [5], and this may in turn cause loss of body condition.

We found the presence of lice and the presence of ear mites to be associated with low BCS. Both ectoparasites are likely to influence the health of the cat [11]. Fleas and ticks were present in low numbers [11] and were not associated with low BCS. It is commonly assumed that high ectoparasite loads on cats that are ill or in poor condition may partly be the result of reduced or insufficient grooming. It is also possible that the higher ectoparasitic loads develop as a result of impaired immunity in animals with poor nutritional status, or that a high ectoparasitic burden is irritating and thus affects the animal’s feeding behaviour, thereby reducing its feed uptake. While no associations between body condition and ectoparasites in cats were found reported in the literature, flea infestation has been associated with season in stray cats [22].

Univariable associations of endoparasites with low BCS were observed, but we could not accurately determine whether age-group, reproductive status or endoparasitic status explained the effect. Infection with *H. taeniaeformis* worms was associated with low BCS. This parasite has generally been considered to have a relatively low impact on cats. However, it is known that *A. abstrusus* can be pathogenic [23], and this supports our finding of an association between the infection and body condition. A similar association was found for *T. cati* egg counts, but not for adult *T. cati*. The eggs are not themselves pathogenic, but they may reflect adult female worm burden. Counts of *A. abstrusus* L1 or *H. taeniaeformis* worms were higher in cats with low BCS, as were juvenile *T. cati* counts.

Although we employed a reasonably large sample, it was still relatively limited for a risk factor study. This can be seen most clearly in connection with FeLV, where only seven cats in total were positive but the prevalence of low BCS among them was 43%. A larger sample size, for which confounding effects could be addressed, is needed to investigate a possible association between this infection and low BCS. Similarly, we would draw attention to the high counts of juvenile *T. cati* worms, which may be associated with low BCS, but this could not be convincingly demonstrated with our sample. An additional limitation with regards to the sample size was the dichotomisation of many findings; for example, we combined loss of a tooth with that of damage to a tooth, irrespective that the effect on BCS could be different, and damage would likely be more severe in terms of its effect on the welfare of the cat. A larger sample size could have prevented this dichotomisation, which was used for many variables.

We cannot be certain that our sample population was representative of the unowned unsocialised domestic cat population. However, since no special criteria were used in constructing the sample, we believe it can be considered representative of the Danish population of unowned unsocialised domestic cats as a whole. It could, however, be the case that healthy cats are reported more often because they might cause more problems as a result of their higher activity level. On the other hand, it is also possible that sick cats are reported more often because people might feel pity for them. With the data we have, we cannot verify which mechanism is more likely, and thus which way the bias could affect the results. Also, neutered cats may be less of an annoyance with less fighting and less mating behaviour, and therefore may be underrepresented. However, we would not expect the proportion of neutered cats to be high in the population because unsocialised cats are usually not released again once caught, owing to Danish legislation. Furthermore, due to practical and logistical constraints, cats were not sampled for an entire season. This precludes interpretation outside the seasons collected.

With the confounding observed with the above-mentioned variables, we can still observe that: Only one juvenile and no neutered cats were observed with a low BCS, and low BCS was primarily observed in spring/summer (April to July), and mostly in intact males or pregnant females, which accounted for 79% of cats with low BCS (Table 2). Consequently, focus could be on intact adults. Season is a given, and we would expect that pregnant cats have a lower BCS due to higher energy expenditure, and neutered cats would experience lower energy expenditure [24]. Also, 79 of the 80 pregnant cats were from the spring/summer–summer/autumn periods, with 8/12 of those with low BCS observed in the spring/summer period. These cats should probably not be identified with health-related problems just because they are pregnant. However, among the 12 pregnant cats with low BCS, 3 (25%) had problems with their teeth, 4 (33%) had lice, 4 (33%) had ear mites, 5 (out of 10, 50%) were positive for *T. gondii*. Among 42 intact male cats with low BCS, 12 (29%) had teeth problems, 10 (24%) had FIV, 13 (out of 40, 33%) had lice and 12 (out of 33, 36%) were positive for *T. gondii*. While it is important to emphasise that causality cannot be inferred with this type of observational data, low BCS in an adult cat could be an indicator of poor health and therefore compromised welfare.

Strengths of the study include its use of a relatively large sample and its investigation of many different variables. The results highlight associations that can be used to define a risk profile.

## Conclusions

A low BCS appears to be a useful indicator of diseases and conditions that may lead to reduced welfare in unowned unsocialised domestic cats. Low BCS was associated with season, age-group and reproduction status (sex, intact status and pregnancy), along with lice and abnormal findings in the GI system. Because season, age-group and reproduction status were associated with other variables such as FIV, skin lesions, tooth lesions and ear mites, the latter may also be underlying causes of low BCS. Assessments of body condition can offer a method of identifying cats that are likely to have pathological conditions and thus compromised welfare, but season, age and reproductive status need to be taken into account when interpreting the observations.

## Data Availability

The datasets used and/or analysed during the current study are available from the corresponding author on reasonable request.

## Abbreviations

BCS: Body condition score
CI: Confidence interval
FeLV: Feline leukaemia virus
FIV: Feline immunodeficiency virus
GI: Gastro-intestinal
NGO: Non-Governmental Organisation
NUTS: Nomenclature of territorial units for statistics
OR: Odds ratio
PCR: Polymerase chain reaction
SCT: Sedimentation and counting technique
Std. error: Standard error
TNR: Trap-neuter-return
